# Identification of prognostic factors for intrahepatic cholangiocarcinoma using long non-coding RNAs-associated ceRNA network

**DOI:** 10.1186/s12935-020-01388-4

**Published:** 2020-07-16

**Authors:** Zhichen Kang, Lixin Guo, Zhuo Zhu, Rongfeng Qu

**Affiliations:** 1 Department of Rehabilitation, the Second Hospital of Jilin University, Changchun, 130022 People’s Republic of China; 2 Department of Anesthesiology, the Second Hospital of Jilin University, Changchun, 130022 People’s Republic of China; 3 Department of Hematology and Oncology, the Second Hospital of Jilin University, No. 218, Ziqiang Street, Changchun, 130022 Jilin People’s Republic of China

**Keywords:** Intrahepatic cholangiocarcinoma, Prognosis, Long non coding RNAs, Competing endogenous RNA network, Sequence Read Archive, Cancer Genome Atlas

## Abstract

**Background:**

Accumulating amount of evidence has highlighted the important roles of long non-coding RNAs (lncRNAs) acting as competing endogenous RNAs (ceRNAs) in tumor pathogenesis. However, the roles of long non coding RNAs (lncRNAs) in the lncRNA-related ceRNA network of intrahepatic cholangiocarcinoma (ICC) still remain enigmatic. The current study aims to identify prognostic factors in the lncRNA-related ceRNA network of ICC.

**Methods:**

The transcriptome sequencing data of lncRNAs, messenger RNA (mRNA) and microRNA (miR) were downloaded from the SRA and TCGA databases. Differentially expressed lncRNAs (DElncRNAs), DEmiRs and DEmRNAs were identified and adopted to construct an lncRNA-miR-mRNA ceRNA network. ICC-associated DEmRNAs were adopted to construct the protein–protein interaction (PPI) network. The expression of the top 6 genes in the hub module was validated with mRNA transcriptome sequencing data and ICC-related gene expression dataset GSE45001, followed by GO and KEGG pathway enrichment analysis. The relationship between the hub gene-associated ceRNA network and the overall survival of patients with ICC was predicted by conducting a Kaplan–Meier survival analysis.

**Results:**

Sixty co-expressed DEmRNAs were identified in the ceRNA network. The top 6 hub genes consisted of downregulated FOS, IGF2, FOXO1 and NTF3, upregulated IGF1R, and insignificantly downregulated HGF in ICC tissues, when compared to that of normal adjacent tissues, followed by the successful construction of lncRNA-miR-hub network consisting of 86 ceRNA modules. MME-AS1 and hsa-miR-182 were associated with overall survival in ICC patients. FOS, IGF1R, IGF2, FOXO1, and NTF3 might target “TGF-β signaling pathway”, “the hedgehog signaling pathway”, “retinol metabolism”, or “type II diabetes mellitus” pathways respectively.

**Conclusion:**

These results indicate that FOS, IGF1R, IGF2, FOXO1, and NTF3 were useful prognostic factors in determining the prognosis of patients with ICC.

## Background

Cholangiocarcinoma is an aggressive malignancy that frequently occurs at the biliary tract, with an unfavorable prognosis [1]. Intrahepatic cholangiocarcinoma (ICC) is rare and is regarded as the least common subtype of cholangiocarcinoma that arises from the epithelial cells of the intrahepatic bile ducts [2]. The dominant risk factors for the pathogenesis of ICC consist of cirrhosis, chronic hepatitis B and C, alcohol use, diabetes, or even obesity [3]. The early combination of different treatment modalities has been proposed to be beneficial for aggressive variants of ICC [4]. However, the longer the time of diagnosis is delayed, the more likely the ICC lesion will undergo a loco-regional extension around the adjacent normal tissues, thus resulting in a poor prognosis [5]. Identifying prognostic factors for ICC is therefore critical for the development of effective treatments for ICC.

The methylation of DLEC1 cilia and flagella associated protein is engaged in a favorable clinical outcome and prognosis in patients with small duct ICC [6]. However, CD90 expression contributed to lymph node metastasis and thereby leads to a poor prognosis in patients with ICC [7]. The expression of VEGFR-3 in ICC also promotes the angiogenesis of lymph, thereby resulting in an unfavorable prognosis [8]. Long non-coding RNAs (lncRNAs) are a multiple class of RNAs engaged in various biological processes [9]. LncRNAs can also serve as both oncogenes and suppressive genes, making them viable targets for tumorigenesis [10]. Competing endogenous RNA (ceRNA) is a newly-emerged regulatory network, on a large scale across the transcriptome with expansion to the genetically functional information in the human genome, which exerts pivotal functions in cancer [11]. Interestingly, ceRNAs indicate a novel modulation on lncRNAs and genes that exert crucial functions on cancer pathogenesis by binding with microRNAs (miRs) in cholangiocarcinoma [12].

In this study, we adopted the transcriptome sequencing data of the lncRNAs, messenger RNA (mRNA) and miR from the Sequence Read Archive (SRA), and Cancer Genome Atlas (TCGA) database, respectively. Differentially expressed lncRNAs (DElncRNAs), DEmiRs and DEmRNAs were identified and adopted to construct an lncRNA-miR-mRNA ceRNA network. ICC-associated DEmRNAs were adopted to construct the protein–protein interaction (PPI) network. The expression of the top 6 genes was identified in the hub module provided by the PPI network, followed by GO and KEGG pathway enrichment analyses. The potential function of genes in the hub module of the ceRNA network was finally subjected to the Gene Set Enrichment Analysis (GSEA). Thus, the purpose of this study was to identify genetic alterations that underlie the prognostic factors of ICC.

## Methods

### Data collection and preprocessing

The original transcriptome sequencing data of the lncRNAs from human ICC and adjacent normal tissues were retrieved until September 2019 from the SRA database (https://www.ncbi.nlm.nih.gov/sra/) and were then subjected to the SRP126672 dataset. The SRP126672 dataset was composed of 30 ICC tissues and 27 adjacent normal tissues. FastQC and Trimmomatic applications were adopted to quality-control and filter the original sequencing data. LncRNAs were quantified based on the Genome Research Project of Encyclopedia of DNA Elements (GENCODE) (GRCh37) catalog (http://www.gencodegenes.org/). The RNA transcriptome sequencing data for ICC were downloaded from TCGA database. MiR sequencing and mRNA sequencing data were downloaded using a data transfer management tool (provided by GDC Apps) (https://tcga-data.nci.nih.gov/). The miR sequencing data and mRNA sequencing data both consisted of 33 ICC tissues and 8 normal tissues, respectively. In addition, the GSE26566 dataset for ICC, which was downloaded from the Gene Expression Omnibus (GEO) database, included 10 samples of ICC and 10 non-tumor liver samples to determine the expression of the hub gene.

### Identification of DEGs

The RNA-seq original data of ICC tissues and adjacent normal tissues were corrected, and normalized, with their expression calculated. DElncRNAs were screened using a DESeq 2 package. The adjusted standard was |log 2 (fold change [FC])| > 2, *p *< 0.05. In addition, the edgeR software package was used to screen DEmiRs and DEmRNAs with thresholds of |log 2 (fold change [FC])| > 2 and FDR < 0.01.

### Construction of lncRNA-miR-mRNA ceRNA network

The ceRNA network was constructed based on the DElncRNAs, DEmiRs and DEmRNAs. The following databases: DElncRNAs, DEmiRs and DEmRNAs, and the miRcode (http://www.mircode.org/) were adopted to predict the markedly downregulated lncRNAs targeted by upregulated miRs and markedly elevated lncRNAs targeted by repressed miRs in ICC. DEGs with the correct trends and targeting relationships served as candidate genes. Next, the TargetScan (http://www.targetscan.org/), miRDB (http://www.mirdb.org/) and miRTarBase online databases (http://mirtarbase.mbc.nctu.edu.tw/php/index.php) were employed to predict markedly downregulated mRNAs targeted by upregulated miRs and markedly elevated mRNAs targeted by repressed miRs in ICC. The mRNAs with the correct trends in intersection among the three databases served as candidate genes. The predicted lncRNA-miR and miR-mRNA were combined to construct the lncRNA-miR-mRNA ceRNA network. Finally, the Cytoscape v3.6.1 software was adopted to visualize and map out the whole constructed network.

### GO and KEGG pathway enrichment analysis

To elucidate the potential biological processes of DEmRNAs related to the ceRNA network in the development of ICC, the DAVID database (https://david.ncifcrf.gov/) was used to perform a GO enrichment analysis of DEmRNAs by setting the default parameters. The GO function, enriched by *p *< 0.05, was considered significant among the available transcriptome sequencing data. In order to understand the potential pathways of DEmRNAs involved in the ceRNA network, the KOBAS database (http://kobas.cbi.pku.edu.cn/index.php) was employed to perform a KEGG pathway enrichment analysis on DEmRNAs, in which the significance of the KEGG pathway was evaluated at *p *< 0.001.

### Construction of the PPI network and module analysis

The interaction between DEGs identified key genes in modules involved in the development of ICC, with a combined score of > 0.4 for a PPI network to be considered as the threshold. The PPI information of DEmRNAs was obtained from the STRING database (http://www.string-db.org/) and a PPI network was subsequently built using the Cytoscape v3.6.1 software. Lastly, the top six key genes in the hub module were obtained from the PPI network using the MCC network topology belonging to the cytoHubba plug-in in Cytoscape.

### Association analysis of hub gene-related network and prognosis of ICC patients

ICC patients were arranged into 2 groups, the high RNA expression group and the low RNA expression group, according to the median expression value of the RNA. Both the Kaplan–Meier method and the log-rank test were used to determine the relationships between DElncRNAs, DEmiRNAs and DEmRNAs (belonging to the ceRNA network), as well as the overall survival (OS) curve of patients. The level of *p* < 0.05 was considered statistically significant.

### Validation of expression of hub genes in ICC

The ICC gene expression dataset GSE45001, from the GEO database, was used to verify the expression of hub genes. The gene expression dataset GSE26566 was also used for the prediction of the hub gene function.

### GSEA of the hub genes in ICC

To better elucidate the potential function of the hub genes in the ceRNA network, a GSEA was performed. According to the median expression of hub genes in the RNA sequencing data, 33 ICC samples from the TCGA database were assigned into the high expression group and the low expression group. The reference gene set is the annotated c2.cp.kegg.v7.0.symbols.gm gene set in the Molecular Signature Database (MSigDB), and the critical criterion was *p *< 0.05.

## Results

### Identification of DEGs between ICC tissues and adjacent normal tissues

**Fig. 1:**
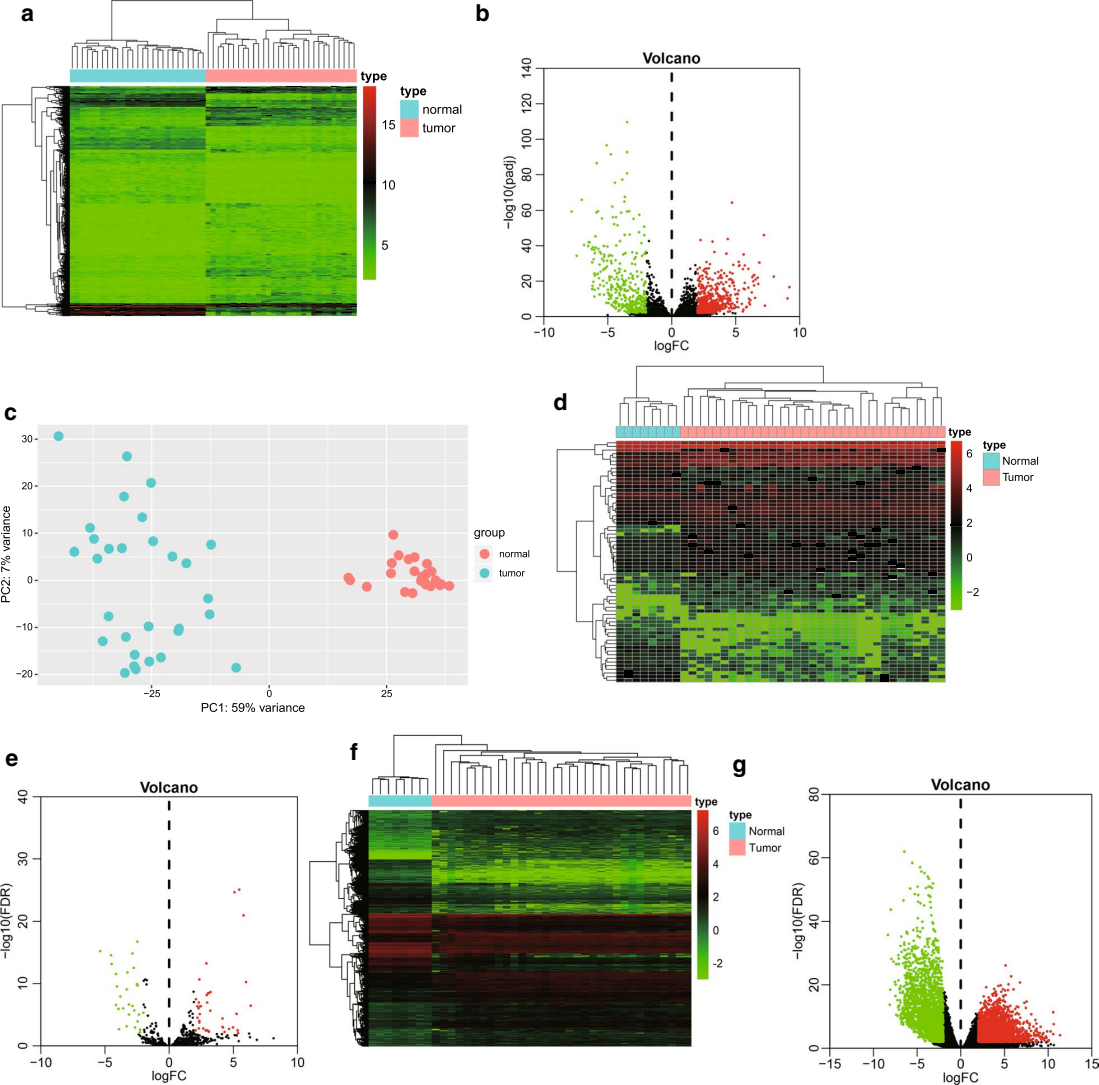
DEGs between ICC tissues and adjacent normal tissues were identified. **a** The heat map of DElncRNAs. The x-axis represents the sample, and the y-axis represents a DElncRNAs. Red represents upregulated DElncRNAs and green represents downregulated DElncRNAs. **b** The volcano plot of DElncRNAs. The y-axis represents the negative base-10 logarithm of the corrected *p* value, and the x-axis represents the base-2 logarithm of FC. Each point represents a DElncRNA. Green dots indicate downregulated DElncRNAs, red dots indicate upregulated DElncRNAs, and black dots indicate lncRNAs that are not differentially expressed. **c** DElncRNAs determined by principal component analysis. Adjacent normal tissues (red, n = 27) and ICC tissues (green, n = 30) were plotted along the axis around the first two main components (PC1 and PC2). **d** The heat map of DEmiRs. The x-axis represents the sample, and the y-axis represents DEmiRs. Red represents upregulated DEmiRs and green represents downregulated DEmiRs. **e** The volcano plot of DEmiRs. The y-axis represents the negative base-10 logarithm of FDR, and the x-axis represents the base-2 logarithm of FC, and each point represents a DEmiR. Green dots indicate downregulated DEmiRs, red dots indicate upregulated DEmiRs, and black dots indicate miRs that are not differentially expressed. **f** The heat map of DEmiRs. The y-axis represents the sample, and the y-axis represents DEmiRs. Red represents upregulated DEmiRs and green represents downregulated DEmiRs. **g** The volcano plot of DEmRNAs. The y-axis represents the negative base-10 logarithm of FDR, the x-axis represents the base-2 logarithm of FC. Each dot represents a DEmRNA. Green dots indicate downregulated DEmRNAs, red dots indicate upregulated DEmRNAs, and black dots indicate mRNAs that are not differentially expressed

To identify the presence of DEmRNAs, DEmiRs and DElncRNAs in ICC, the SRP126672 dataset of RNA-seq sequencing data, composed of 30 ICC tissues and 27 adjacent normal tissues, was downloaded from SRA database. FastQC and Trimmomatic applications were used to quality-control and filter the original sequencing data, and the DESeq 2 package was used to screen DElncRNAs. A total of 912 DElncRNAs were obtained, specifically with 524 upregulated and 388 downregulated DElncRNAs (Fig. 1a, b). The different expression of DElncRNAs could help distinguish ICC tissues and adjacent normal tissues based on principal component analysis (Fig. 1c). DEmiR and DEmRNA sequencing data (counts) of ICC and adjacent normal tissues were obtained from the TCGA database, followed by differential analysis using the edgeR package. Finally, 66 DEmiRs (38 upregulated and 28 downregulated) (Fig. 1d, e) and 5522 DEmRNAs (3158 upregulated and 2364 downregulated) (Fig. 1f, g) were acquired.

### The lncRNA-miR-mRNA ceRNA network in ICC

To better understand the role of lncRNAs and miRs in the ceRNA network of ICC tissues, a lncRNA-miR-mRNA-ceRNA network was established. Initially, 66 DElncRNAs targeted by 66 DEmiRs was retrieved from miRcode database to obtain 31,452 lncRNA-miR pairs. We then screened the significantly downregulated lncRNAs targeted by upregulated miRs and obviously upregulated lncRNAs targeted by downregulated miRs in ICC. Then, only DEGs with the correct trends and targeting relationships served as candidate genes. The selected candidate genes contain 66 pairs of lncRNA-miR, including 18 markedly up-regulated DElncRNAs, 19 down-regulated DElncRNAs, 10 significantly up-regulated miRs, and 2 significantly down-regulated miRs.

**Fig. 2:**
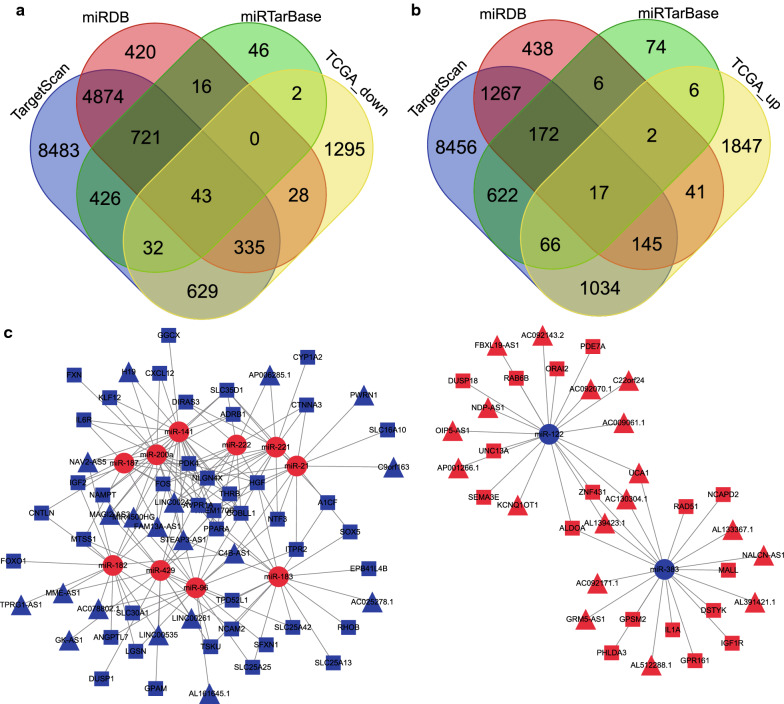
Diagram showing the interactions of the lncRNA-miR-mRNA ceRNA network. **a** The downstream targets by upregulated DEmiRs. The four colored ovals represent the prediction results of the TargetScan, miRDB and miRTarBase databases and the 2364 significantly down-regulated DEmRNAs obtained by the differential analysis, respectively. The middle part represents the intersection of the four sets of data. **b** The downstream targeted by downregulated DEmiRs. The four colored ovals represent the prediction results of the TargetScan, miRDB, and miRTarBase databases, and 3158 significantly up-regulated DE mRNAs obtained by differential analysis, respectively. The middle part represents the intersection of the four sets of data. **c** Construction of ceRNA network of lncRNA-miR-mRNA in ICC. The network consists of 37 lncRNA nodes, 12 miR nodes, and 60 mRNAs. The triangle represents lncRNA, the circle represents miR, and the square represents mRNA. Nodes highlighted in red and blue indicate upregulation and downregulation, respectively

In addition, 16457, 7052, and 2081 target mRNAs were predicted from the following databases: TargetScan, miRDB, and miRTarBase respectively. The 10 downregulated candidate DEmiRs were intersected with 2364 downregulated DEmRNAs, yielding 43 shared genes (Fig. 2a), and 2 downregulated candidate DEmiRs were intersected with 3158 upregulated DEmRNAs, yielding 17 share genes (Fig. 2b). The shared genes served as candidate genes and were applied to generate 136 miR-mRNA pairs including 43 significantly down-regulated and 17 significantly up-regulated DEmRNAs. A total of 37 DElncRNAs, 12 DEmiRs, and 60 DEmRNAs were used to construct the ceRNA network. Based on the aforementioned lncRNA-miR pairs and miR-mRNA pairs, a ceRNA network consisting of 37 lncRNA nodes, 12 miR nodes, and 60 mRNA nodes in ICC was constructed (Fig. 2c).

### GO and KEGG pathway enrichment analysis on DEmRNAs

**Fig. 3:**
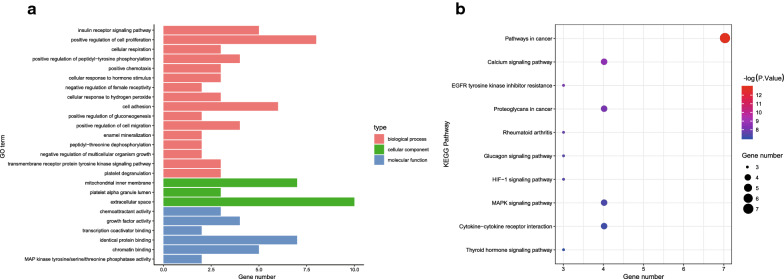
GO and KEGG pathway enrichment analysis were conducted on DEmRNAs **a** The 60 DEmRNAs enriched with GO terms were obtained using the DAVID database (*p *< 0.05). The y-axis represents GO terms, and the x-axis represents number of DEmRNAs enriched with GO terms. **b** The 60 DEmRNAs enriched KEGG pathways obtained from KOBAS database (*p *< 0.001). The y-axis represents KEGG pathways, and the x-axis represents number of DEmRNAs enriched on the pathway. The larger bubbles indicate the increased number of enriched DEmRNAs, and the increased saturations of red color represent more significantly enriched DEmRNAs

To identify the biological functions and pathways of the 60 DEmRNAs in the ceRNA network, we used the DAVID database to perform a GO function enrichment analysis and the KOBAS database for KEGG pathway enrichment analysis. GO enrichment analysis indicated that DEmRNAs related to biological processes were mainly enriched in GO terms including the following: insulin receptor signaling pathway, positive regulation of cell proliferation, cell respiration and other items (*p *< 0.05). DEmRNAs that were related to cellular components had a close resemblance with mitochondrial inner membrane (*p *< 0.05). DEmRNAs that were related to molecular functions were mainly enriched in GO terms of chemoattractant activity, growth factor activity, and transcriptional coactivator binding (*p *< 0.05) (Fig. 3a). The KEGG analysis revealed that DEmRNAs were significantly enriched in pathways such as cancer pathways, calcium signaling pathways, cytokin-cytokine receptor interactions, MAPK singling pathway, and proteoglycans in cancer (*p *< 0.001) (Fig. 3b). Coherently, 60 significantly different DEmRNAs exerted a pivotal role in the occurrence and development of ICC by the mediation of the above biological processes.

### Identification of 6 hub genes in PPI network

**Fig. 4:**
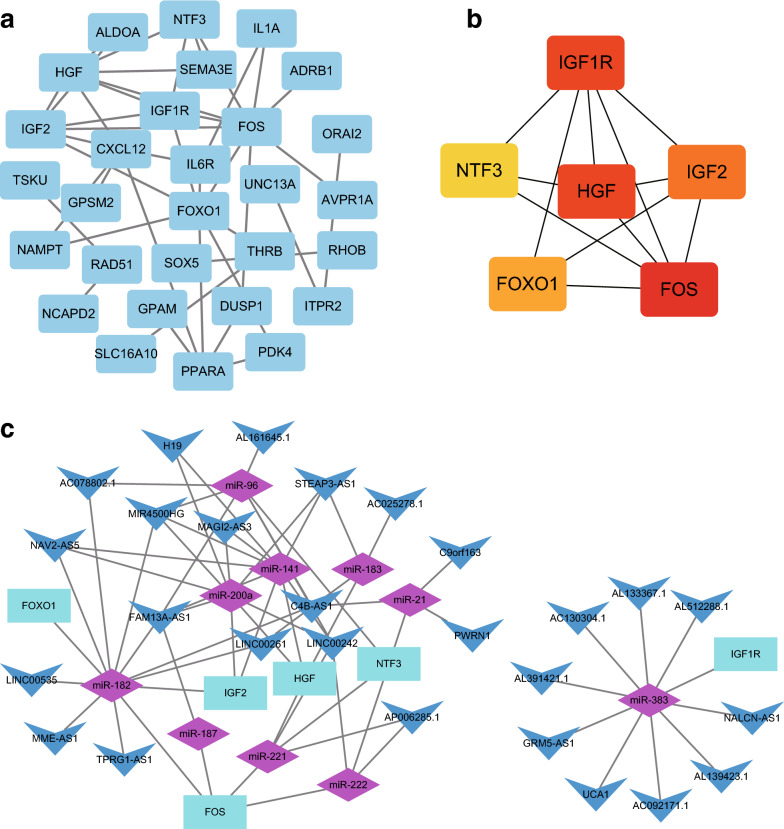
Hub genes identified in the PPI network. **a** PPI network of 60 DEmRNAs, consisting of 60 nodes and 41 edges. **b** The first 6 genes from hub modules among the 60 DEmRNAs in PPI network. The node color gradually changes from yellow to red based on the log (2) fold change. **c** lncRNA-miR-hub gene network. The network consists of 27 lncRNAs, 10 miRs, and 6 genes in the hub model. Vs indicate lncRNA, diamond indicates miR, and rectangle indicates mRNA

The PPI network was constructed based on DEmRNAs, comprising of 60 nodes and 41 edges (Fig. 4a). To identify the top six hub genes in the PPI network, the MCC network topology in the cytoHubba plug-in was used to determine the relationship of DEmRNAs. Then, 6 hub genes were obtained, including Fos proto-oncogene, AP-1 transcription factor subunit (FOS), insulin like growth factor 1 receptor (IGF1R), hepatocyte growth factor (HGF), insulin like growth factor 2 (IGF2), forkhead box O1 (FOXO1), and neurotrophin 3 (NTF3) (Fig. 4b). Finally, the lncRNA-miR-hub gene subnetwork was constructed (Fig. 4c), including 86 ceRNA regulatory modules.

### Association of hub gene-associated ceRNA network in ICC with OS of patients

**Fig. 5:**
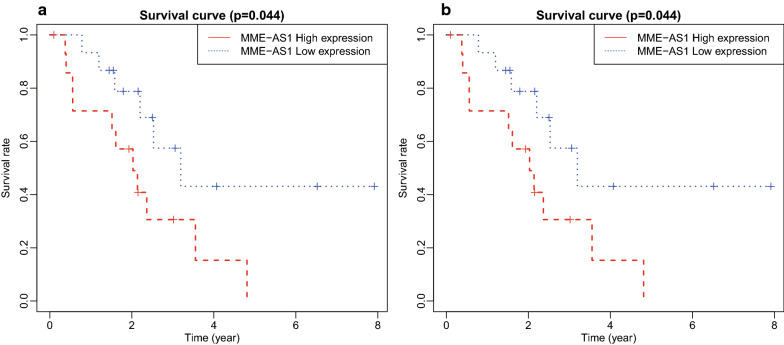
Hub gene-associated ceRNA network in ICC was associated with OS of patients. **a** Kaplan–Meier survival curve of DElncRNA MME-AS1 in ICC. The abscissa represented the survival time of patients with ICC, and the ordinate represented survival rate of patients with ICC. **b** Kaplan–Meier survival curve of DEmiRNA hsa-miR-182 in ICC. The abscissa represented survival time of patients with ICC, and the ordinate represents survival rate of patients with ICC

To identify the relationships of 6 reliable hub genes (FOS, IGF1R, HGF, IGF2, FOXO1, and NTF3), and associated DElncRNAs and DEmiRNAs with OS in patients with ICC, a Kaplan–Meier curve analysis was performed. The results indicated that only DElncRNA MME-AS1 and DEmiRNA hsa-miR-182 were correlated with OS of patients with ICC (Fig. 5a, b). Moreover, the high expression of MME-AS1 was inversely correlated with the OS rate of ICC patients, whereas the high expression of hsa-miR-182 was positively correlated with the OS rate of ICC patients, and MME-AS1 could target and orchestrate hsa-miR-182 (Fig. 5c). MME-AS1 and miR-182 have not been reported in ICC according to literature, but miR-182 can inhibit cell proliferation of various cancers, such as ovarian cancer and colorectal cancer [13, 14], suggesting that MME-AS1 may affect the prognosis of patients with ICC by targeting hsa-miR-182.

### Validation of FOS, HGF, IGF2, FOXO1, NTF3 and IGF1R expression in ICC

**Fig. 6:**
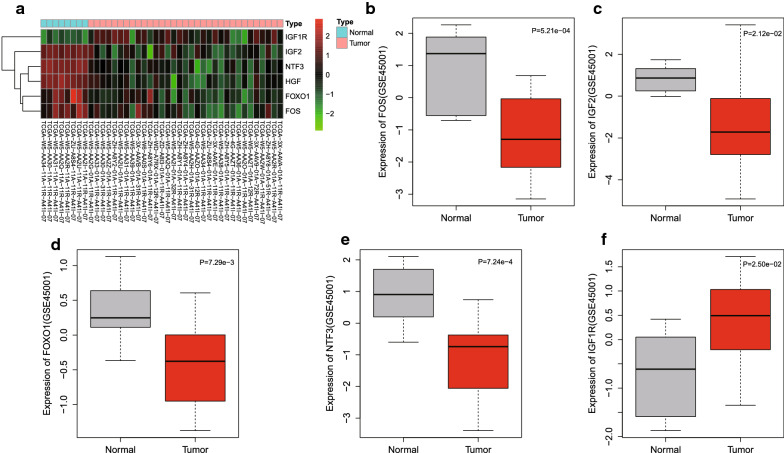
The expressions of FOS, HGF, IGF2, FOXO1, NTF3 and IGF1R were validated in ICC. **a** The heat map of genes in the hub module expression in ICC tissues and adjacent normal tissues from the TCGA database. The x-axis represents the sample, and the y-axis represents the genes in the hub module. Red represents the up-regulated genes and green represents the down-regulated genes. **b**–**f** The expressions of FOS, IGF2, FOXO1, NTF3, and IGF1R in the gene expression dataset, GSE45001, of ICC

To better validate our analysis, the expression of 6 hub genes were evaluated in 33 ICC tissues and 8 adjacent normal tissues. The results (Fig. 6a) demonstrated that FOS, HGF, IGF2, FOXO1, and NTF3 were remarkably downregulated while IGF1R expression was notably elevated in ICC tissues (|logFC| > 2, FDR < 0.01) compared to that of adjacent normal tissues. To verify the accuracy of the analysis results, we also validated the expression of FOS, HGF, IGF2, FOXO1, NTF3, and IGF1R in the GSE45001 ICC dataset, and found that FOS, IGF2, FOXO1, and NTF3 were markedly poorly expressed, IGF1R was highly expressed, and HGF was slightly poorly expressed in ICC (Fig. 6b–f). Thus, HGF was excluded from the following analysis. The above findings showed that the results of our analysis have certain reliability.

### GSEA prediction of biological pathways related to hub genes in ICC

**Fig. 7:**
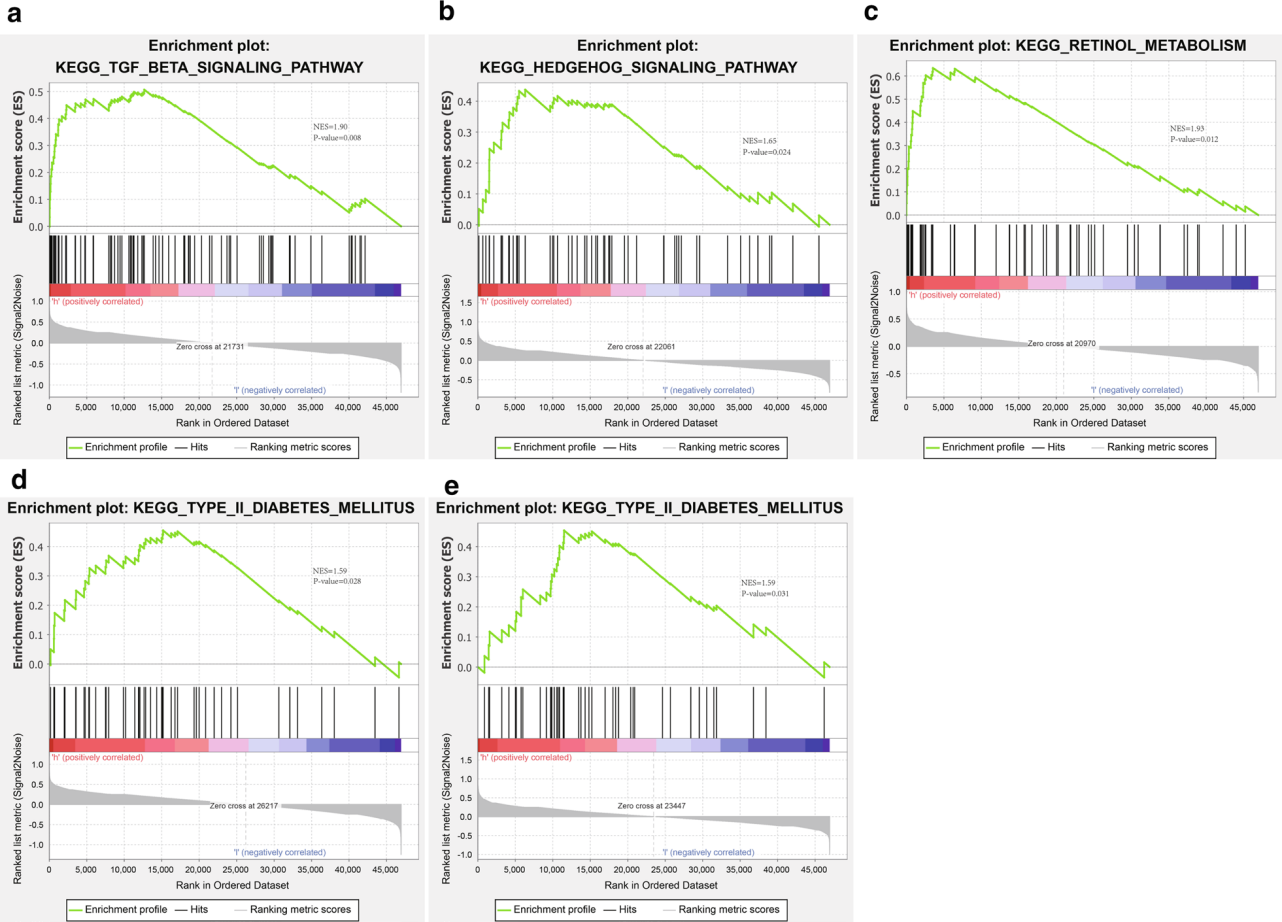
Biological pathways associated with 5 hub genes were predicted in ICC tissues using GSEA. **a** The first functional gene set enriched in the FOS high expression group. **b** The first functional gene set enriched in the IGF2 high expression group. **c** The first functional gene set enriched in the IGF1R high expression group. **d** The first functional gene set in the FOXO1 high expression group. **e** The first functional gene set in the NTF3 high expression group

To better understand the biological pathways related to FOS, IGF1R, IGF2, FOXO1, and NTF3, we classified 33 ICC samples from the TCGA database into the high expression group and the low expression group. A GSEA was conducted along with the annotated c2.cp.kegg.v7.0.symbols.gmt gene set in the MSigDB as the reference gene set. Based on the obtained results, the ICC tissues in the FOS, IGF1R, and IGF2 high expression groups were significantly enriched in “the TGF-β signaling pathway”, “the hedgehog signaling pathway”, and “the retinol metabolism”, respectively (Fig. 7a–c); the ICC tissues in the FOXO1 and NTF3 high expression groups were notably enriched on the “type 2 diabetes mellitus” (Fig. 7d, e). As previously reported, TGF-β is one of the main signaling pathways promoting the progression of cholangiocarcinoma. The inhibition of the TGF-β signaling pathway can induce the anti-proliferation properties of cholangiocarcinoma cells [15, 16]. Additionally, it was also documented that the inhibition of the hedgehog signaling pathway, a potential therapeutic target for human cholangiocarcinoma, attenuated the progression of carcinogenesis in vitro and subsequently increased the necrosis rate of cholangiocarcinoma [17, 18]. Retinol metabolism has been less studied in cholangiocarcinoma, but there is literature suggesting that retinol metabolism is a key pathway in the development of cholangiocarcinoma [19]. Moreover, diabetes is associated with an increased risk of ICC, showing similar etiologies [20–22]. However, other prospective studies are still required to validate the aforementioned associations. In summary, it was suggested that five key genes in ICC may exert effects through the TGF-β signaling pathway, the hedgehog signaling pathway, retinol metabolism, and type 2 diabetes mellitus.

## Discussion

The present study is still in its preliminary research phase pertaining to the prognostic factors for patients with ICC. To gain more insight into the molecules involved in the prognosis of patients with ICC, we analyzed the transcriptome sequencing data of the lncRNAs, mRNA and miR to construct a lncRNA-miR-mRNA ceRNA network, where 60 co-expressed DEmRNAs associated with ICC were identified. The main notable findings in the current study are that the expression of FOS, IGF2, FOXO1, and NTF3 was diminished, but the expression of IGF1R was enhanced in ICC tissues, compared with that of normal adjacent tissues. In addition, these five hub genes might regulate the development of ICC by targeting the “TGF-β signaling pathway”, “the hedgehog signaling pathway”, “retinol metabolism”, or “type II diabetes mellitus”.

FOS proteins are characterized by a leucine zipper motif and a basic region with a helix-turn-helix motif that binds to DNA and serve as an oncogene and as transcription factors by binding on the DNA sequences [23]. The expression of FOS has been proposed to be positively related with the expression of c-Myb in colorectal cancer cells, while the expression of c-Myb is repressed in colorectal cancer tissues, suggesting that expression of FOS is also downregulated in colorectal cancer tissues [24], which is concordant with the current study. The elevation of FOS-like antigen 1 expression is positively correlated with the progression of perihilar cholangiocarcinoma [25]. More importantly, a prior research illustrated that FOS was involved in gene expression regulated by TGF-β [26]. Furthermore, the tumor-promoting effects of TGF-β signaling pathway have been reported in cholangiocarcinoma [15].

IGFs (IGF1 and IGF2) expedite glucose metabolism with their availability modulated by IGF binding proteins, and function as prognostic factors for type 1 diabetes [27]. The induction of IGF2 is also partially involved in the proliferation and survival of rhabdomyosarcoma cells [28]. Interestingly, IGF2 has been reported to be methylated in ICC compared to extrahepatic cholangiocarcinoma [29]. IGF2 has been elucidated to be associated with retinol metabolism [30]. Furthermore, Liu et al. observed that retinol metabolism was implicated in cholangiocarcinoma development [19].

IGF1 maintains the phenotype of the tumor and allows the transformed murine pheochromocytoma cells to survive [31]. The inhibition of IR/IGF1R reduced the epithelial-mesenchymal transition and cancer stem cell-like traits in ‘resistant cells’ of cholangiocarcinoma [32]. The elevated expression of IGF1R was observed in tumor necrosis factor-related apoptosis-inducing ligand (TRAIL)-resistant gastric cancer cells, thus enhancing TRAIL resistance in gastric cancer cells [33]. According to genes encoding proteins related to insulin receptors, IGF1R is able to stimulate renal cancer cells [34]. It has been elucidated that IGFIR activates the Hedgehog signaling pathway in growth-plate (GP) chondrocytes [35]. Research conducted by Guo et al. revealed that the activation of the Hedgehog signaling pathway is involved in proliferation, migration and EMT progression of cholangiocarcinoma cells [36].

FOXO1 transcription factors orchestrate various cell types that are important in the host response [37]. Moreover, FOXO1 has been described to assume a pivotal role in tumor initiation, progression and metastasis [38]. A prior research indicated that the downregulation of FOXO1 elevated tumorigenesis and invasion of prostate cancer cells [39]. FOXO1 expression was reduced in patients with type II diabetes mellitus, and that the downregulation of FOXO1 induces insulin resistance states that qualitatively and quantitatively mimic the function of adipocytes from patients with type II diabetes mellitus [40]. NTF3, belonging to the neurotrophic factor family which encompasses nerve growth factor, brain-derived neurotrophic factor, and neurotrophic factor 4/5, has become a key mediator of neuronal development in early neurogenesis and throughout adulthood [41]. Moreover, the correlation between NTF3 and diabetes mellitus has been identified [42]. It has been shown that insulin resistance is a main determinant of the carcinogenic effect of type II diabetes mellitus [43]. Notably, Lee et al. elaborated that diabetes mellitus was a risk factor for ICC [44]. In fact, a literature reported that type II diabetes mellitus could increase the risk of ICC by 80%, and that the increase in ICC incidence and mortality observed over the past 3 decades was similar to that of type II diabetes mellitus and metabolic syndrome [22].

## Conclusion

In conclusion, our research identified several novel genetic alterations and pathways associated with the prognosis of ICC, as well as the potential roles of miRs, lncRNAs and mRNAs in the development of ICC via bioinformatic analysis. Based on the ceRNA network, we also discovered that FOS, IGF1R, IGF2, FOXO1, and NTF3 might target the “TGF-β signaling pathway”, “the hedgehog signaling pathway”, “retinol metabolism”, or “type II diabetes mellitus” pathways respectively, thereby modulating the subsequent development of ICC. A substantial insight gained from the understanding of the molecules involved in the prognosis of ICC contributed to an increased efficacy of the available treatments for patients with ICC. However, further studies with more selective candidate genes and a larger sample size are required to clearly define their roles in the development of ICC. Additional studies also need to be performed to examine the underlying mechanisms of FOS and TGF-β signaling pathway.

## Data Availability

Data sharing not applicable to this article as no datasets were generated or analyzed during the current study.

## Abbreviations

ceRNAs: Competing endogenous RNAs
lncRNAs: Long non-coding RNAs
ICC: Intrahepatic cholangiocarcinoma
DElncRNAs: Differentially expressed lncRNAs
GENCODE: Genome Research Project of ENCyclopedia of DNA Elements
TCGA: The Cancer Genome Atlas
GEO: Gene Expression Omnibus
